# Exploring anti-malarial potential of FDA approved drugs: an in silico approach

**DOI:** 10.1186/s12936-017-1937-2

**Published:** 2017-07-18

**Authors:** Gayatri Ramakrishnan, Nagasuma Chandra, Narayanaswamy Srinivasan

**Affiliations:** 1 Indian Institute of Science Mathematics Initiative, Indian Institute of Science, Bangalore, 560012 India; 2 Department of Biochemistry, Indian Institute of Science, Bangalore, 560012 India; 3 Molecular Biophysics Unit, Indian Institute of Science, Bangalore, 560012 India; 4 Division of Genetics, Department of Medicine, Brigham and Women’s Hospital and Harvard Medical School, Boston, MA 02115 USA

**Keywords:** Antimalarial agents, Drug repurposing, Drug targets, *Plasmodium falciparum*, Sequence analysis

## Abstract

**Background:**

The critically important issue on emergence of drug-resistant malarial parasites is compounded by cross resistance, where resistance to one drug confers resistance to other chemically similar drugs or those that share mode of action. This aspect requires discovery of new anti-malarial compounds or formulation of new combination therapy. The current study attempts to contribute towards accelerating anti-malarial drug development efforts, by exploring the potential of existing FDA-approved drugs to target proteins of *Plasmodium falciparum*.

**Methods:**

Using comparative sequence and structure analyses, FDA-approved drugs, originally developed against other pathogens, were identified as potential repurpose-able candidates against *P. falciparum.* The rationale behind the undertaken approach is the likeliness of small molecules to bind to homologous targets. Such a study of evolutionary relationships between established targets and *P. falciparum* proteins aided in identification of approved drug candidates that can be explored for their anti-malarial potential.

**Results:**

Seventy-one FDA-approved drugs were identified that could be repurposed against *P. falciparum.* A total of 89 potential targets were recognized, of which about 70 are known to participate in parasite housekeeping machinery, protein biosynthesis, metabolic pathways and cell growth and differentiation, which can be prioritized for chemotherapeutic interventions. An additional aspect of prioritization of predicted repurpose-able drugs has been explored on the basis of ability of the drugs to permeate cell membranes, i.e., lipophilicity, since the parasite resides within a parasitophorous vacuole, within the erythrocyte, during the blood stages of infection. Based on this consideration, 46 of 71 FDA-approved drugs have been identified as feasible repurpose-able candidates against *P. falciparum,* and form a first-line for laboratory investigations. At least five of the drugs identified in the current analysis correspond to existing antibacterial agents already under use as repurposed anti-malarial agents.

**Conclusions:**

The drug-target associations predicted, primarily by taking advantage of evolutionary information, provide a valuable resource of attractive and feasible candidate drugs that can be readily taken through further stages of anti-malarial drug development pipeline.

**Electronic supplementary material:**

The online version of this article (doi:10.1186/s12936-017-1937-2) contains supplementary material, which is available to authorized users.

## Background

Over the past decade, extensive measures have been undertaken to prevent or cure malaria, resulting in reduction of mortality rates by 60% [1]. Despite the tremendous progress, malaria continues to be a global burden with 214 million new incidences and about 0.4 million deaths in the year 2015 alone, the primary cause of which is the emergence of drug-resistant strains of *Plasmodium falciparum.* The current anti-malarial regimens include several drugs administered either alone or in combination, which can be grouped based on their drug class, as summarized in Table 1. Albeit moderately effective, the current treatment regimens remain inadequate due to sub-optimal safety profile in some cases, as described in Table 1, and mainly due to emergence of drug-resistant parasites. Anti-malarial drug resistance, mainly contributed by transporters in the parasite [2], is also compounded by ‘cross resistance’, where resistance to one drug confers resistance to other chemically similar drugs or those that share mode of action. The current attempt to prevent development of resistance to artemisinin, a potent first-line anti-malarial agent, includes administration of artemisinin-based combination therapy (ACT) [1], i.e., artemisinin with non-artemisinin partners, such as amodiaquine and mefloquine (Table 1). However, resistance to almost all known anti-malarial agents has reportedly emerged [3, 4] limiting combinatorial therapy as well. This necessitates the discovery and development of either new anti-malarial agents or unexplored combination of drugs that may not only reduce mortality and morbidity of the disease, but also reduce the risk of resistance to anti-malarial drugs.

**Table 1:** Details on current anti-malarial agents

Drug name	Drug class	Anti-malarial activity	Side effects
Quinine	Cinchona alkaloids	Accumulates in food vacuoles and forms toxic haem complexes	Side effects include hearing impairment, rashes, vertigo, vomiting and in some cases neurotoxicity
Quinidine			
Mefloquine	Quinolines and derivatives		Nausea, dizziness, diarrhoea, bradycardia and neurotoxicity
Chloroquine			May cause psoriasis
Amodiaquine			Vomiting, dizziness and in some cases hepatic disorders
Primaquine		Believed to block oxidative metabolism in the parasite	Anorexia, vomiting, cramps and anaemia
Halofantrine	Phenanthrenes and derivatives	Causes parasite membrane damage by forming cytotoxic complexes	Nausea, diarrhoea, itching and high cardiotoxicity
*Sulfadoxine*	Benzene and substituted derivatives	Inhibit synthesis of folates	Skin reactions (rare)
*Sulfamethoxypyridazine*			
Proguanil			Very few: hair loss and mouth ulcers
Pyrimethamine	Diazines		Occasional rashes
*Tetracycline*	Tetracyclines	Inhibits translation	–
*Doxycycline*			Depression of bone growth and gastrointestinal disturbances
*Clindamycin*	Carboxylic acids and derivatives	Inhibits protein synthesis	Nausea, vomiting and cramps
*Azithromycin*	Macrolides and analogues		May cause angioedema and jaundice
Artemisinin	Lipids and lipid-like molecules	Believed to affect mitochondrial electron transport chain [46] or disrupt cellular redox cycling or inhibition of haem metabolism [47]	Nausea, anorexia, dizziness and neurotoxicity
Atovaquone	Naphthalenes	Affects mitochondrial electron transport chain	May cause rashes, diarrhoea and headache

DrugBank (v.4.3) [26]. The drugs highlighted in italics denote anti-bacterials repurposed for use against malaria

Identifying new therapeutic uses of existing drugs has become an attractive practicable strategy due to significant advantages in cost and time involved in the drug development pipeline. Indeed, this strategy has been exploited previously, which led to the formulation of anti-malarial treatment regimens that include anti-bacterials such as sulfonamides, tetracyclines, clindamycin and azithromycin [5]. Albeit successful earlier as a part of combination therapy, these anti-bacterials have become inefficient due to emergence of drug-resistant parasites. Recently, high-throughput chemical library screening initiatives have been useful in identification of repurpose-able drugs that await evaluation on their anti-malarial efficacy [6, 7]. With the lack of adequate anti-malarial treatment regimen and increasing incidences of drug-resistant parasites, exploring existing drugs for use against malaria is gaining rapid importance [8]. The current study makes an effort to contribute towards the same with the use of FDA-approved drugs.

Using the concept of within-target-family selectivity of small molecules [9], i.e., the likeliness of a small-molecule to bind to related targets, potential repurpose-able drug candidates against malaria could be recognized by means of exploration of evolutionary relationships between targets of FDA-approved drugs and *P. falciparum* proteins. An initial filter to exclude drugs known to target human proteins aided in elimination of drugs with undesirable pharmacological potential. This drug-target identification methodology facilitated recognition of 89 *P. falciparum* proteins which could serve as targets for 71 FDA-approved drugs. The drug-target associations thus predicted can serve as a useful resource for experimental endeavours in the light of anti-malarial drug development and drug discovery.

## Methods

### Dataset

Information on gene products encoded in the genome of *P. falciparum* 3D7 isolate was obtained from PlasmoDB (release 24) [10]. Proteins annotated as pseudogenes were excluded as such entries correspond to genes that apparently lack protein-coding ability and are unlikely to be expressed in the cell. The protein sequences corresponding to 5400 protein-coding genes were thus retrieved from the database. Furthermore, protein expression profiles of the parasite proteins during their developmental stages in human were extracted from the published works of Pease et al. [11], Florens et al. [12] and Le Roch et al. [13] for intra-erythrocytic stages (ring, trophozoite, schizont, merozoite), from Lindner et al. [14] for liver stage (sporozoite), and from Silvestrini et al. [15] for the final gametocyte stage. Details on FDA-approved drugs (1634) and the associated protein targets (1573) were obtained from DrugBank (version 4.3), an extensive resource on drug and target information.

### Approach to recognize potential drug-target interactions

The protocol adopted to identify FDA-approved drugs that can be repurposed against proteins in *P. falciparum* is similar to the protocol employed in an earlier published report on repurposing drugs against *Mycobacterium tuberculosis* H37Rv [16]. The exclusion of drugs known to be effective against human proteins and the exclusion of anti-malarial agents currently in use (Table 1) formed the initial step of the approach. Also, only those drugs that hold evidence on pharmacological validation against established targets were considered. Thus, 196 FDA-approved drugs associated with 138 protein targets were considered for subsequent analysis. The likelihood of drugs to be active as potential anti-malarial agents is dependent on the similarity of known targets to *P. falciparum* proteins. To recognize target-related proteins in *P. falciparum,* an iterative sequence search program, jackhammer availed through HMMER3.0 suite [17], was employed at an E-value cut-off of 0.0001 and five rounds of iteration. An alignment coverage cut-off of 70% or an alignment encompassing at least one functional and/or structural domain was used as an additional criterion to eliminate unreliable hits characterized by short stretches of alignment.

Once reliable target-related proteins are recognized in *P. falciparum*, a comparative structural analysis was undertaken to pinpoint probable drug-binding sites in the parasite proteins, based on information on ligand-binding sites in the known targets. Structural information for target proteins and parasite proteins was obtained from PDB (Protein Data Bank). For parasite proteins of no known structure, structural models were retrieved from ModBase [18] or built using MODELLER v.9.14 [19]. The structural models obtained were checked for reliability based on z-DOPE (Discrete Optimized Protein Energy) score [20] (<0), ModPipe Quality Score (MPQS) cut-off of 1.1 and query coverage threshold of 80% or a query coverage of at least one functional and/or structural domain. Comparative evaluation of binding sites across established targets and their homologues in *P. falciparum* was pursued depending on the availability of crystal structures of ligand-bound targets. A structural alignment algorithm, TM-align [21], was employed to assess the extent of conservation of residues in local aligned regions between the parasite proteins and corresponding related targets. A TM-score cut-off of 0.5 was used, which typically implies convincing structural similarity of the proteins aligned [22]. In certain cases, a reliable structural model for the *P. falciparum* protein or its domain could not be built, primarily due to abundance of low-complexity regions in the protein and/or presence of non-conserved inserts within the functional domains. Indeed, these features contribute to the pronounced sequence divergence observed in genome of *P. falciparum* [23–25]. Thus, in such cases, the aligned regions of interest between the protein sequences of *P. falciparum* and related targets were manually examined to infer feasibility of drug-target interactions in *P. falciparum*. Similarly, in the absence of crystal structures of established targets, potential drug-target interactions in the parasite proteins were conjectured on the basis of alignment of functionally relevant regions.

## Results

*Plasmodium falciparum* homologues of established targets of FDA-approved drugs were identified using sequence and structural analyses described in “Methods”. If *P. falciparum* homologue of a known drug target could be identified, the drug concerned is an attractive possibility for anti-malarial activity.

### Potential repurpose-able drug candidates

**Fig. 1:**
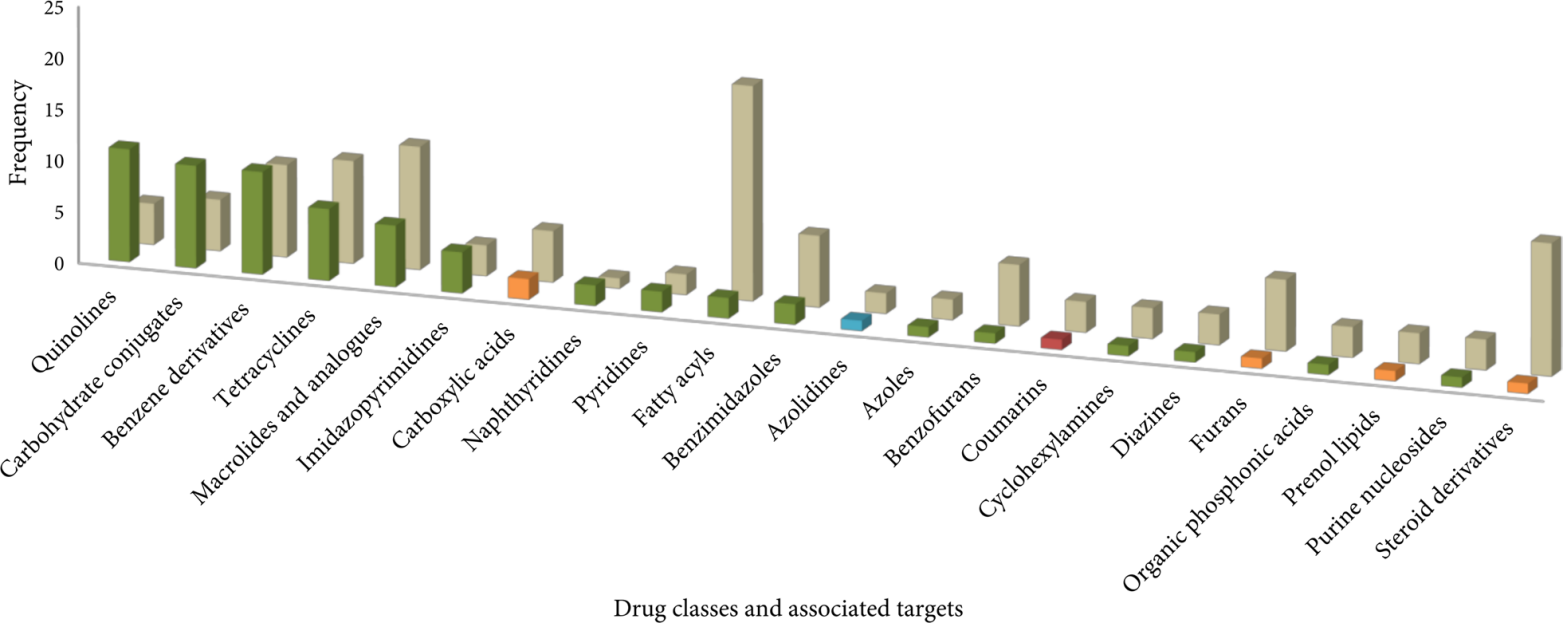
Distribution of drug-target associations in the context of drug classes. The distribution of 71 drugs, identified in the study, according to their drug classes is represented in the form of a *bar chart* (front); the *bars* are *coloured* based on the organisms the drugs are known to be active against: antibacterial (*green*), antiviral (*orange*), anthelmintic (*blue*), and antifungal (*red*). A bar graph representation of the number of *P. falciparum* targets (*light brown*) predicted to be associated with each drug class is also shown

A total of 71 FDA-approved drugs, predominantly constituted by antibacterial agents (56), were recognized as repurpose-able candidates against 89 *P. falciparum* proteins. The complete list of predicted drug-target associations is provided in Additional file 1. At least five of the anti-malarial drugs listed in Table 1 are anti-bacterial and have been repurposed for use against malaria. Based on hierarchical, rule-based, structural classification of drugs, as obtained from DrugBank [26], the 71 drugs could be grouped into 22 drug classes. Figure 1 outlines the distribution of 71 drugs under the 22 classes, along with number of predicted parasite targets associated with each drug class. The predominance of antibacterial agents, represented as green coloured bars, can be readily inferred from the Figure. Notably, a number of instances can be observed in the Figure, which corresponds to drugs with polypharmacological potential, i.e., the ability to act on multiple targets. The most striking example in the Figure is that of two antibacterial drugs (DB00410: mupirocin, DB00548: azelaic acid) under the class ‘Fatty acyls’ which have been predicted to target 21 parasite proteins; of which nine parasite proteins involved in aminoacylation are potential targets for mupirocin and 12 metabolic proteins of the parasite are potential targets for azelaic acid.

Azelaic acid is a naturally occurring compound produced by *Malassezia furfur,* and is approved for use as a topical antibacterial agent. This drug is believed to inhibit several metabolic enzymes in bacteria, including tyrosinase, mitochondrial enzymes of the respiratory chain, thioredoxin reductase, 5-α-reductase and DNA polymerase. Indeed, the 12 potential targets recognized in *P. falciparum* belong to these categories of oxido-reductive enzymes, four of which are localized in a vital organelle of the parasite, the apicoplast (highlighted in Table 2). Considering the ability to target multiple metabolic enzymes, azelaic acid forms an attractive repurpose-able candidate against *P. falciparum.* Such polypharmacological drugs have the ability to stand tolerance against development of drug resistance in malarial parasites, and indeed are of immense interest in rationalizing anti-malarial drug discovery and development efforts. Table 2 summarizes the details on the predicted targets of azelaic acid.

**Table 2:** Summary of predicted drug-target associations for two FDA-approved drugs, azelaic acid and mupirocin

Gene ID	Protein description	Expression of proteins in different developmental stages						UniProt ID of related target	Protein description	Source organism	Seq. Identity (%)	Structural similarity, TM-score (cut-off >0.5)	Associated drug
		Sp	R	T	Sc	M	G						
PF3D7_0311200	Valine-tRNA ligase	✓	✓	✓	✓	✓	×	P41972	Isoleucine-tRNA ligase	*Staphylococcus aureus*	20	0.82	DB00410: Mupirocin
PF3D7_0622800	Leucine-tRNA ligase	×	✓	✓	×	✓	×	P41972	Isoleucine-tRNA ligase	*Staphylococcus aureus*	20	0.64	DB00410: Mupirocin
PF3D7_0828200	Leucine-tRNA ligase	✓	×	✓	×	✓	✓	P41972	Isoleucine-tRNA ligase	*Staphylococcus aureus*	20	0.68	DB00410: Mupirocin
PF3D7_1005000	Methionine-tRNA ligase	✓	✓	✓	×	✓	✓	P41972	Isoleucine-tRNA ligase	*Staphylococcus aureus*	22	0.97	DB00410: Mupirocin
PF3D7_1015200	Cysteine-tRNA ligase	✓	✓	✓	✓	✓	✓	P41972	Isoleucine-tRNA ligase	*Staphylococcus aureus*	22	0.80	DB00410: Mupirocin
PF3D7_1034900	Methionine-tRNA ligase	✓	✓	✓	✓	✓	×	P41972	Isoleucine-tRNA ligase	*Staphylococcus aureus*	24	0.77	DB00410: Mupirocin
PF3D7_1225100	Isoleucine-tRNA ligase	✓	×	✓	✓	✓	✓	P41972	Isoleucine-tRNA ligase	*Staphylococcus aureus*	29	0.97	DB00410: Mupirocin
PF3D7_1332900	Isoleucine-tRNA ligase	✓	✓	✓	✓	✓	✓	P41972	Isoleucine-tRNA ligase	*Staphylococcus aureus*	28	0.78	DB00410: Mupirocin
PF3D7_1461900	Valine-tRNA ligase	✓	✓	✓	✓	✓	✓	P41972	Isoleucine-tRNA ligase	*Staphylococcus aureus*	25	0.75	DB00410: Mupirocin
PF3D7_0203900	5′-3′ exonuclease, N-terminal resolvase-like domain	×	×	✓	✓	×	×	P19821	DNA polymerase I	*Thermus aquaticus*	25	0.88	DB00548: Azelaic acid
PF3D7_0616800	Malate:quinone oxidoreductase	✓	×	✓	✓	✓	✓	P66010	Thioredoxin reductase	*Staphylococcus aureus*	21	0.61	DB00548: Azelaic acid
PF3D7_0625300	DNA polymerase I	×	×	✓	✓	×	✓	P00582	DNA polymerase I	*Escherichia coli*	23	0.93	DB00548: Azelaic acid
PF3D7_0720400	Ferridoxin reductase-like	✓	×	✓	✓	✓	✓	P66010	Thioredoxin reductase	*Staphylococcus aureus*	20	0.64	DB00548: Azelaic acid
PF3D7_0815900	Lipoamide dehydrogenase	✓	✓	✓	×	×	✓	P66010	Thioredoxin reductase	*Staphylococcus aureus*	22	0.67	DB00548: Azelaic acid
PF3D7_0915000	Type-II NADH:ubiquinone oxidoreductase	×	×	✓	✓	✓	✓	P66010	Thioredoxin reductase	*Staphylococcus aureus*	20	0.63	DB00548: Azelaic acid
PF3D7_0923800	Thioredoxin reductase	×	×	✓	✓	×	✓	P66010	Thioredoxin reductase	*Staphylococcus aureus*	24	0.67	DB00548: Azelaic acid
PF3D7_1034400	Flavoprotein subunit of succinate dehydrogenase (SDHA)	✓	×	✓	✓	✓	✓	P66010	Thioredoxin reductase	*Staphylococcus aureus*	23	0.62	DB00548: Azelaic acid
PF3D7_1232200	Dihydrolipoyl dehydrogenase, mitochondrial	✓	✓	✓	✓	✓	✓	P66010	Thioredoxin reductase	*Staphylococcus aureus*	21	0.67	DB00548: Azelaic acid
PF3D7_1411400	Plastid replication-repair enzyme (PREX)	✓	×	✓	✓	✓	✓	P00582	DNA polymerase I	*Escherichia coli*	25	0.83	DB00548: Azelaic acid
PF3D7_1419800	Glutathione reductase	✓	×	✓	✓	✓	✓	P66010	Thioredoxin reductase	*Staphylococcus aureus*	23	0.63	DB00548: Azelaic acid
PF3D7_1435300	NAD(P)H-dependent glutamate synthase	✓	×	✓	✓	✓	✓	P66010	Thioredoxin reductase	*Staphylococcus aureus*	24	0.65	DB00548: Azelaic acid

The different developmental stages of the parasite during its lifecycle in human are represented as: *Sp* sporozoite, *R* ring, *T* trophozoite, *Sc* schizont, *M* merozoite, *G* gametocyte. Tick symbols indicate up-regulated expression of the protein

**Fig. 2:**
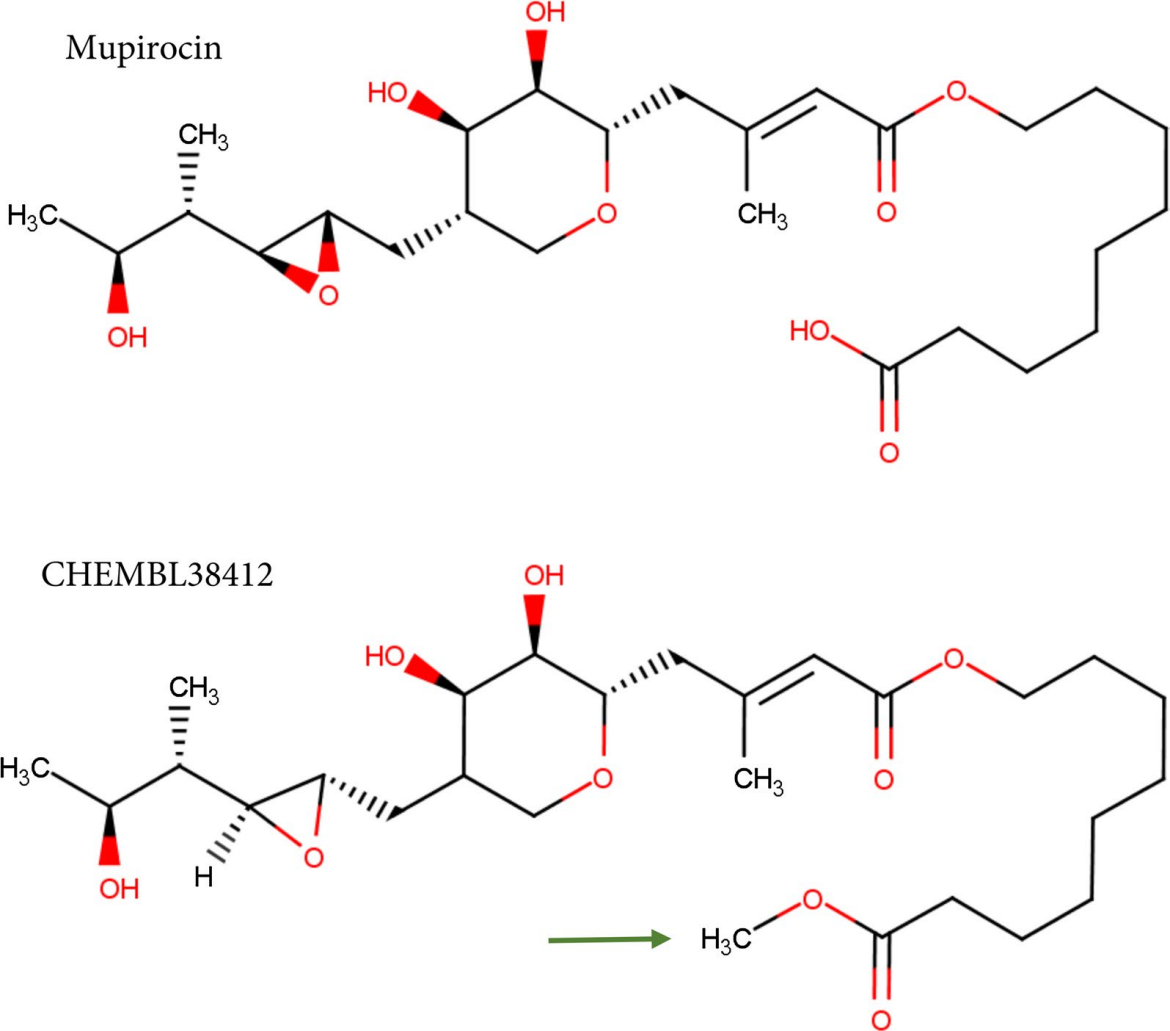
Mupirocin and its analogue. Two-dimensional chemical representations of the drug mupirocin and its analogue CHEMBL38412 is shown. The presence of non-hydrolysable ester group at C1 position in CHEMBL38412 is depicted with an *arrow* in *green*. This figure was generated using Marvin 15.5.18, 2015, ChemAxon [45]

Mupirocin, an antibiotic isolated from *Pseudomonas fluorescens,* is one of the widely used bacteriocidal agent known to inhibit isoleucyl-tRNA synthetase (also known as isoleucyl-tRNA ligase). Recently, a study demonstrated the potent inhibitory activity of mupirocin against blood-stage *P. falciparum* [27]. Despite its effectiveness, mupirocin is unfit to serve as a lead for anti-malarial therapy as it is restricted to topical usage due to rapid hydrolysis in body fluids. Nevertheless, non-hydrolysable mupirocin analogues with evidence on inhibitory activity can be explored for their potential use against *P. falciparum*. Indeed, two analogues of mupirocin, CHEMBL38412 and CHEMBL315230, were identified from an earlier published study [16] where two-step in silico screening (two-dimensional followed by three-dimensional molecular similarity calculations) was employed on a set of FDA-approved drugs against a database of ChEMBL compounds [28, 29], which hosts exhaustive details on drug-like bioactive compounds largely extracted from literature and other databases. Of the two mupirocin analogues CHEMBL38412 and CHEMBL315230, which are experimentally verified inhibitors of isoleucyl-tRNA synthetase [30, 31], CHEMBL38412 is reportedly a non-hydrolysable analogue of mupirocin and exhibits similar antibacterial activity in vitro as mupirocin [30]. Elucidations on binding site and binding pose of CHEMBL38412 can be readily inferred based on structural similarity between mupirocin-bound isoleucyl-tRNA synthetase and related parasite proteins (Table 2 and Additional file 2: Figure S1) and the extent of molecular similarity between the drug and its analogue as illustrated in Fig. 2. Based on reasonable structural similarity amongst related proteins as well as the compounds, the mupirocin analogue CHEMBL38412 forms an attractive lead and thus can be investigated further for its anti-malarial activity.

Several other drug classes corresponding to drugs with possible polypharmacological potential, as delineated in Fig. 1, can be investigated further. Figure 1 also illustrates populated drug classes including ‘Quinolines’, ‘Carbohydrate conjugates’, ‘Benzene derivatives’ and ‘Imidazopyrimidines’ with relatively fewer predicted targets associated with them. Indeed, this observation implies the possibility of combination therapy. But the risk of cross-resistance exists, where resistance to one drug may confer resistance to other chemically similar drugs or those drugs that share similar mechanism of action. Reports on tetracycline and doxycycline resistance, alone or in combination with other drugs, limits the possibility of using approved drugs chemically similar to tetracycline. Seven such drugs (DB00595: oxytetracycline, DB01301: rolitetracycline, DB00618: demeclocycline, DB00256: lymecycline, DB00453: clomocycline, DB01017: minocycline, DB00560: tigecycline) in the drug class ‘Tetracyclines’ were recognized in the current analysis as potential drug candidates against ten potential parasite targets (Fig. 1). Thorough experimental investigations are necessary to ascertain the likelihood of these drugs as repurpose-able candidates.

### Drug targets recognized in *Plasmodium falciparum*

**Fig. 3:**
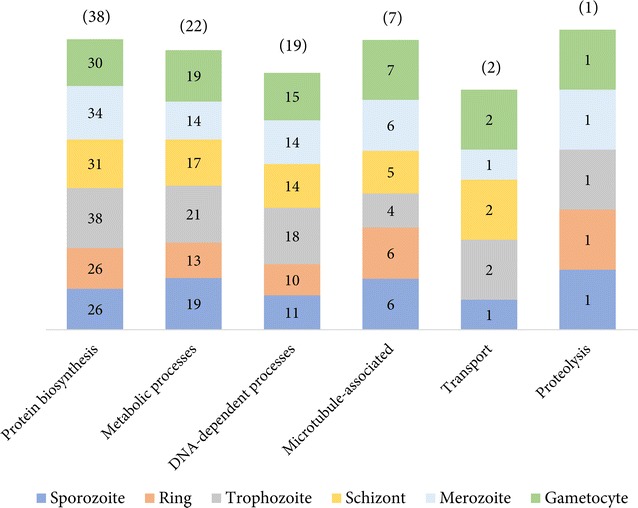
Functional relevance of the predicted targets. The distribution of 89 potential targets under six functional categories expressed in six life-cycle stages is represented as a stacked *bar chart*. The absolute number of proteins under each functional category is indicated in *brackets*, while the numbers in stacked *bars* denote the number of proteins, which are upregulated in a life-cycle stage in each functional category

The 89 potential targets identified in *P. falciparum* could be categorized under six functional classes, including protein biosynthesis, metabolic processes, DNA-dependent activities, microtubule-associated processes, transport and proteolytic activities, most of which are expressed in all six life-cycle stages of infection in human host. Figure 3 illustrates the distribution of predicted targets across the six life-cycle stages of *P. falciparum* binned under six functional categories. As shown in Fig. 3, 38 of 89 predicted targets participate in cellular protein synthesis, which are predominantly expressed in all six life-cycle stages of the parasite. This category includes proteins participating in aminoacylation and protein translation, essential for housekeeping activities. Other proteins of parasite housekeeping machinery including DNA polymerases, DNA topoisomerases, DNA gyrases, microtubules and structural constituents of cytoskeleton, qualify as attractive targets as these *P. falciparum* proteins expressed in all six life-cycle stages during infection in humans play a significant role in regulating rate of translation, cell growth and cell development [12, 23]. Thus, targeting such parasite proteins (64) belonging to the functional classes ‘Protein biosynthesis’, ‘DNA-dependent processes’ and ‘Microtubule-associated’ (Fig. 3) can impede growth and transition between different developmental stages of the parasite. Based on the current analysis, a list of 46 drugs were identified, most of which are antibacterial agents, predicted to target 64 proteins involved in parasite housekeeping machinery (Additional file 1).

**Fig. 4:**
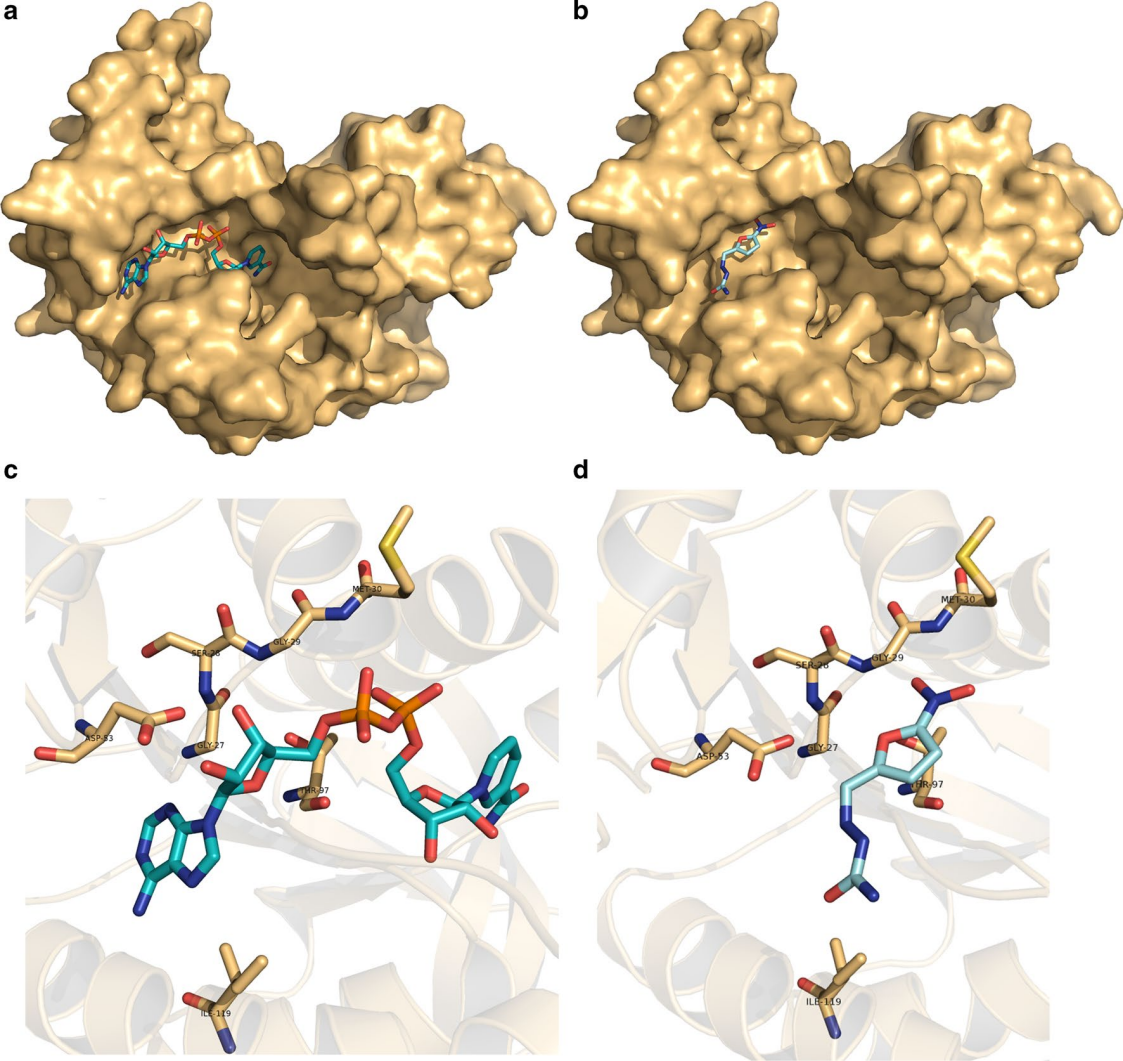
Targeting *pf*LDH. **a** Surface representation of crystal structure of *pf*LDH bound with its native substrate NADH, represented as sticks, is shown (PDB:1LDG). **b** Surface representation of crystal structure of *pf*LDH with docked nitrofural, in sticks, is shown. The details of the residues of *pf*LDH involved in interaction with its native substrate are illustrated in **c** and with the nitrofural in **d**. For convenience, only those residues are shown in stick representation which are common to binding substrate and the predicted drug

An important characteristic of *P. falciparum* is its ability to undergo vast re-organization of metabolic make-up during the course of its life cycle in multiple host environments. Inhibition of critically important metabolic enzymes can be detrimental for the parasite’s survival, one of which is the glycolytic enzyme lactate dehydrogenase (*pf*LDH) essential for intra-erythrocytic growth of the parasite [32], recognized as a target for the drug nitrofural (DB00336) in the current analysis. Studies on *pf*LDH have reported a distinct nature of NADH binding pocket [32] where the anti-malarial chloroquine is believed to bind competitively [33]. Nitrofural, a potent antibacterial agent, is known to inhibit several bacterial enzymes involved in aerobic and anaerobic degradation of glucose and pyruvate. Crystal structure of nitrofural-bound nitroreductase enzyme in *Escherichia coli* demonstrated the accommodation of the drug in the cofactor binding site [34]. Thus, the cofactor binding site in the crystal structure of *pf*LDH, i.e., the NADH binding pocket, along with adjacent solvent exposed cavities, were used as receptor grids to dock nitrofural using Glide (Grid-based ligand docking with energetics) [35–37] and assess the predicted binding energy and binding pose of the approved drug. Using Glide XP (extra precision) scoring function [37], the predicted binding site of the drug nitrofural was evaluated. Figure 4 illustrates the docked pose of nitrofural, achieved with a best possible energy score of −6.97 kcal/mol, at the NADH binding pocket of *pf*LDH. The docked drug was identified to make contacts with critical residues of *pf*LDH that are involved in cofactor binding, including Asp53 and Thr97 (Fig. 4c, d), implying the likeliness of nitrofural in inhibiting the glycolytic enzyme of the parasite. In addition to *pf*LDH, six *P. falciparum* metabolic proteins, including malate dehydrogenase (PF3D7_0618500), lactate dehydrogenase (PF3D7_132520), lipoamide dehydrogenase (PF3D7_0815900), thioredoxin reductase (PF3D7_0923800), dihydrolipoyl dehydrogenase (PF3D7_1232200), and glutathione reductase (PF3D7_1419800), several of which are essential for the parasite’s growth during intra-erythrocytic development stages, were recognized as potential targets for nitrofural. Considering such a polypharmacological potential of nitrofural, further experimental investigations on this FDA-approved drug can reveal insights into its usefulness as an anti-malarial agent. Other metabolic enzymes identified as targets include enoyl-acyl carrier reductase, dihydropteroate synthetase, thioredoxin reductase, bifunctional dihydrofolate reductase–thymidylate synthase, and other oxido-reductive enzymes which have been regarded as attractive targets for anti-malarial drug development [38], many of which are expressed in all six stages of the parasite’s life cycle (Fig. 3). The identification of such pharmaceutically relevant metabolic proteins justifies the strength of the approach used and thus, the feasibility of approved drugs predicted to target such proteins can be further investigated.

**Fig. 5:**
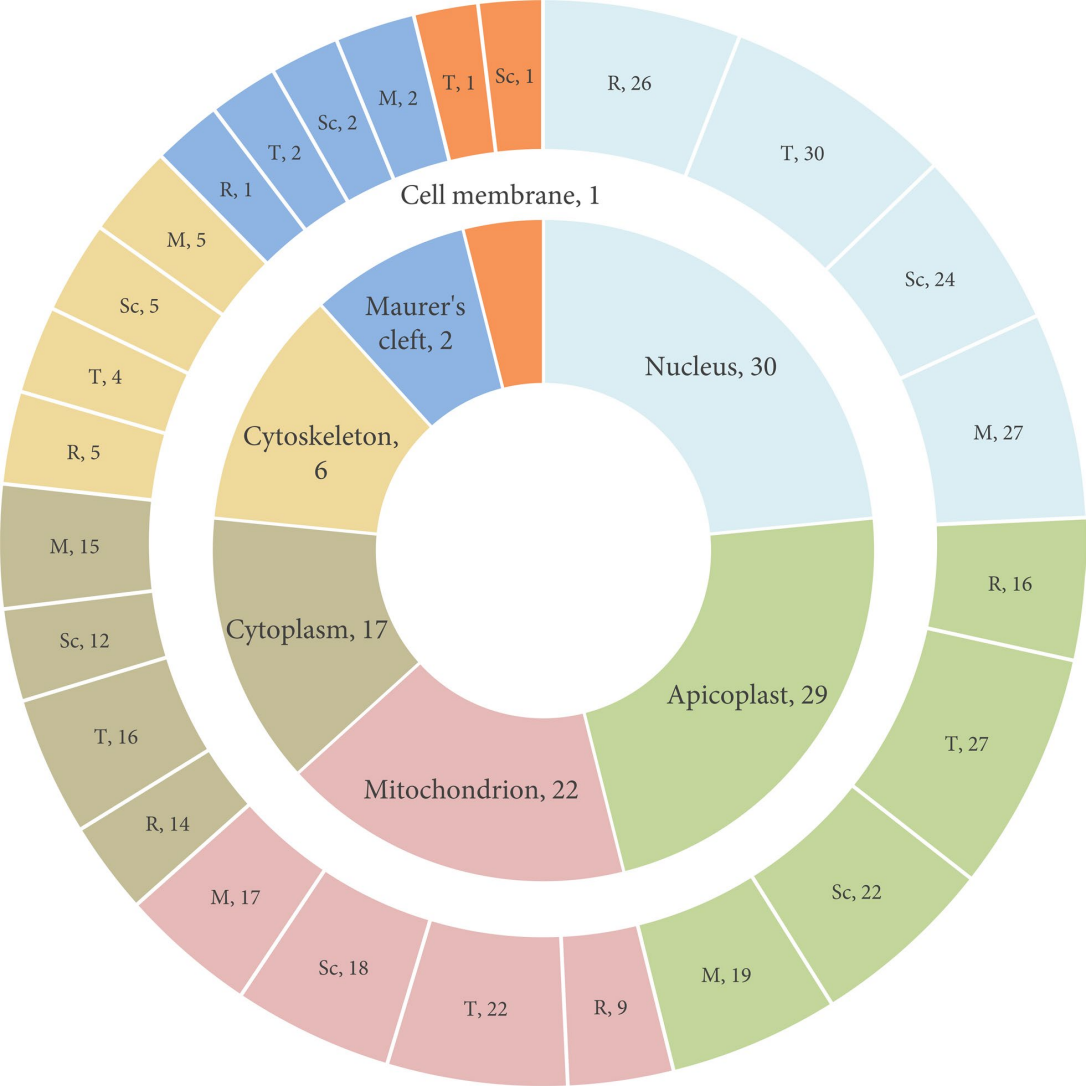
Subcellular localization of predicted targets. The distribution of predicted targets based on their subcellular localization is delineated in the inner ring. The outer ring denotes number of associated proteins with up-regulated expression in each of the intra-erythrocytic stages, i.e., *R* ring, *T* trophozoite, *Sc* schizont and *M* merozoite

*Plasmodium falciparum* comprises a relict-plastid, termed as the apicoplast, which is indispensable for the parasite’s growth and typically contains a range of metabolic pathways and housekeeping processes that differ from those in human hosts [39]. Thus, parasite proteins pertaining to the apicoplast present ideal strategies for drug design. Of 89 predicted targets, 29 were identified as constituents of apicoplast—8 metabolic enzymes and 21 involved in housekeeping machinery. These entries are highlighted in Additional file 1. Information on subcellular localization of other parasite proteins was obtained from Malaria Metabolic Pathways database [40]. Figure 5 illustrates the distribution of number of predicted targets in terms of their subcellular localization in *P. falciparum* (inner ring), along with information on upregulated, intra-erythrocytic stage-specific expression of associated proteins (outer ring). As shown in Fig. 5, more than half of the predicted *P. falciparum* targets are localized in subcellular organelle: nucleus, apicoplast and mitochondrion.

While recognition of proteins essential for the growth and survival of the parasite as targets gains significance, targeting such proteins may be challenging as the parasite resides within the erythrocytes, and additionally, within a protective encasing of parasitophorous vacuole, during blood stages of infection. To reach a target localized in an organelle in the parasite during its intra-erythrocytic development stages, a drug is required to permeate at least four membranes: erythrocyte cell membrane >parasitophorous vacuole membrane >*P. falciparum* cell membrane >organellar membrane. It is thus of importance to consider lipophilicity of the drugs, i.e., the ability of the compounds to dissolve in lipophilic or non-aqueous solutions, which in turn determines the ADMET (absorption, distribution, metabolism, excretion, toxicity) properties of the drug and the likelihood of its therapeutic success [41]. Lipophilicity is typically measured in terms of logarithm of partition coefficient (logP), which is the ratio of concentrations of a compound between two phases:non-aqueous:octanol and aqueous:water. According to Lipinski’s rule of five [42], logP value of an active compound should be no more than five, i.e., a concentration difference of 1:100,000 between water and octanol phases. A desirable compound should neither be too lipophilic (logP > 5) nor be too hydrophilic (low or negative logP), for adequate absorption or permeation across cellular membranes. Information on experimentally determined logP values of 71 FDA-approved drugs was obtained from DrugBank. Forty-six of 71 drugs were recognized which satisfied the logP value criterion in an optimal range of (0–5). These drugs are speculated to be active against *P. falciparum* during the symptomatic blood stages of infection, and therefore can be prioritized to formulate chemotherapeutic regimen against malaria. Table 3 summarizes the details of 46 FDA-approved drugs that have the potential to influence anti-malarial drug development. Several of the 46 practicable set of drugs, approved for use against other pathogenic infections, have been exploited for their use as anti-malarial agents, examined either in vitro or in vivo (Table 3). Such concurring instances justify the strength of the protocol followed.

**Table 3:** Details on 46 FDA-approved drugs that are likely to be active against *Plasmodium falciparum* during the course of infection

Drug name	DrugBank ID	Category	Drug class	LogP
DB00199	Erythromycin [48]	Antibacterial	Macrolides and analogues	3.06
DB00218	Moxifloxacin	Antibacterial	Quinolines	2.9
DB00250	Dapsone [49]^a^	Antibacterial	Benzene derivatives	0.97
DB00256	Lymecycline	Antibacterial	Tetracyclines	0.3
DB00263	Sulfisoxazole [50]	Antibacterial	Benzene derivatives	1.01
DB00336	Nitrofural	Antibacterial	Furans	0.23
DB00400	Griseofulvin	Antifungal	Benzofurans	2.18
DB00410	Mupirocin [27]	Antibacterial	Fatty acyls	2.45
DB00426	Famciclovir	Antiviral	Imidazopyrimidines	0.6
DB00446	Chloramphenicol [51]^b^	Antibacterial	Carboxylic acids	1.14
DB00453	Clomocycline	Antibacterial	Tetracyclines	0.2
DB00487	Pefloxacin [52]	Antibacterial	Quinolines	0.27
DB00518	Albendazole [53]	Anthelmintic	Benzimidazoles	2.7
DB00537	Ciprofloxacin [52]	Antibacterial	Quinolines	0.28
DB00548	Azelaic acid	Antibacterial	Fatty acyls	1.57
DB00560	Tigecycline [54]	Antibacterial	Tetracyclines	0.8
DB00567	Cephalexin [55]	Antibacterial	Carboxylic acids	0.65
DB00576	Sulfamethizole	Antibacterial	Benzene derivatives	0.54
DB00609	Ethionamide	Antitubercular	Pyridines	0.5
DB00615	Rifabutin [56]	Antitubercular	Macrolactams	4.1
DB00618	Demeclocycline	Antibacterial	Tetracyclines	0.2
DB00730	Thiabendazole [53]	Anthelmintic	Benzimidazoles	2.47
DB00817	Rosoxacin	Antibacterial	Quinolines	3.0
DB01015	Sulfamethoxazole [57]	Antibacterial	Benzene derivatives	0.89
DB01017	Minocycline [55]	Antibacterial	Tetracyclines	0.05
DB01044	Gatifloxacin [58]	Antibacterial	Quinolines	2.6
DB01045	Rifampicin [56]	Antitubercular	–	2.7
DB01051	Novobiocin [52]	Antibacterial	Coumarins	4.1
DB01137	Levofloxacin [58]	Antibacterial	Quinolines	2.1
DB01155	Gemifloxacin	Antibacterial	Naphthyridines	2.3
DB01165	Ofloxacin [52]	Antibacterial	Quinolines	0.65
DB01208	Sparfloxacin [58]	Antibacterial	Quinolines	2.5
DB01220	Rifaximin	Antibacterial	Macrolactams	2.6
DB01256	Retapamulin	Antibacterial	Prenol lipids	4.37
DB01298	Sulfacytine	Antibacterial	Benzene derivatives	0.055
DB01321	Josamycin	Antibacterial	Macrolides and analogues	3.22
DB01361	Troleandomycin	Antibacterial	Macrolides and analogues	4.3
DB01581	Sulfamerazine	Antibacterial	Benzene and derivatives	0.14
DB01582	Sulfamethazine	Antibacterial	Benzene and derivatives	0.89
DB01603	Meticillin	Antibacterial	Lactams	1.22
DB01764	Dalfopristin [59]	Antibacterial	Macrolide lactams	1.58
DB02703	Fusidic acid [60]	Antibacterial	Steroid derivatives	4.42
DB04576	Fleroxacin	Antibacterial	–	0.24
DB06729	Sulfaphenazole	Antibacterial	Azoles	1.52
DB06771	Besifloxacin	Antibacterial	Quinolines	0.54
DB08604	Triclosan [61]	Antibacterial	Quinolines	4.98

The references included in 23 entries are with respect to the reports on experimental evidence of anti-malarial activity of the drugs

^a^Chloroproguanil–dapsone combination drug Lapdap has been withdrawn from the market after the observation on significant reductions in haemoglobin levels of patients with glucose-6-phosphate dehydrogenase deficiency [62]

^b^Chloramphenicol is known to be associated with adverse haematological side-effects [63]

The predictions were compared with previously published studies on recognizing anti-malarial potential of approved drugs [8, 43]. Nine drug-target associations were identified pertaining to apicoplast proteins and anti-tubercular drugs (rifamixin, rifampicin) concurring with the predictions made in the current study which, in addition to the set of drugs mentioned in Table 3, can be readily taken through further stages of anti-malarial drug development pipeline.

## Conclusion

An established concept of within-target-family selectivity of small molecules has been pursued which led to the recognition of 71 FDA-approved drugs that can be repurposed against 89 *P. falciparum* proteins. The framework on drug-target identification methodology established in an earlier published study [16], served as a guiding tool for the workflow adopted. An initial step on exclusion of drugs active against human proteins, minimize the chance of obtaining drugs of undesirable pharmacological potential. Recognition of polypharmacological drugs, capable of targeting multiple proteins, are of great interest for parasites such as *P. falciparum,* as such drugs can stand tolerance against development of drug resistance. Indeed, the majority of the drugs identified have the potential to target multiple parasite proteins. About 70 of 89 potential targets identified in the current analysis, are known to participate in parasite housekeeping machinery, protein biosynthesis, metabolic pathways, and cell growth and differentiation. The drug-target associations corresponding to such parasite targets of interest can be investigated further for therapeutic relevance. While the approach undertaken provides a set of promising drug candidates and potential *P. falciparum* targets, it is limited in terms of evaluation of quantitative drug-target selectivity. Investigations focused on pharmacokinetics and pharmacodynamics of drugs thus form a pre-requisite to assess the risk of possible drug-target toxicity in humans, in addition to investigations on feasibility of existing drugs as potential anti-malarials.

Considering the intra-erythrocytic growth of *P. falciparum* during the course of infection, an attempt has been made to prioritize the predicted repurpose-able FDA-approved drugs on the basis of their ability to permeate cell membranes, i.e., lipophilicity, since the parasite resides within a parasitophorous vacuole in the erythrocyte. Using experimentally determined values of octanol–water partition coefficient of drugs, it is estimated that 46 of 71 drugs are feasible repurpose-able candidates against *P. falciparum.* These approved drugs can be readily pursued further for an experimental follow-up in vivo.

Much of the drugs identified in the study as prospective anti-malarial agents are antibiotics. The anti-malarial potential of antibiotics has been well-documented in literature [44], several of which require to be administered in multiple doses in combination with other drugs. Owing to their unintended impact on host microbiome, use of multi-dose antibiotics perhaps should be restricted to patients with severe complications due to malaria and must only be considered after carefully weighing results from preclinical investigations and clinical trials. On similar lines, in the context of safety concerns, clues on FDA-approved drugs obtained from the prioritization exercise attempted in this study can be validated through experimental investigations for efficacy, toxicity, safety, and for the promising candidates, also the therapeutic potential.

The present study provides a set of FDA-approved drugs that are likely to target proteins of the malarial parasite. Albeit, consideration of existing approved drugs can be time and cost-efficient in terms of accelerating anti-malarial drug development efforts, it is extremely important to realize that drug safety cannot be assumed for generic drugs. Indeed, it is imperative that safety of drugs for re-use as anti-malarial agents must be re-assessed and concluded with a careful assessment of risk–benefit ratio for a given indication and duration of treatment.

## Additional files

**Additional file 1.** Details on drug-target associations predicted for *Plasmodium falciparum.*

**Additional file 2.** Additional Figure.

## Abbreviations

ACT: artemisinin-based combination therapy
ADMET: absorption, distribution, metabolism, excretion and toxicity
DOPE: discrete optimized protein energy
Glide: grid-based ligand docking with energetics
MPQS: ModPipe quality score
PDB: protein data bank
*pf*LDH: lactate dehydrogenase of *P. falciparum*
