# Supplementary material for: Exploring anti-malarial potential of FDA approved drugs: an in silico approach
Source: Malar J. 2017 Jul 18;16:290. doi: 10.1186/s12936-017-1937-2 (PMC5516367; doi:10.1186/s12936-017-1937-2)
Supplement: Supplementary file 2 — Additional file 2. Additional Figure. [file 12936_2017_1937_MOESM2_ESM.docx]

**Additional file 2**

**Additional Figure**


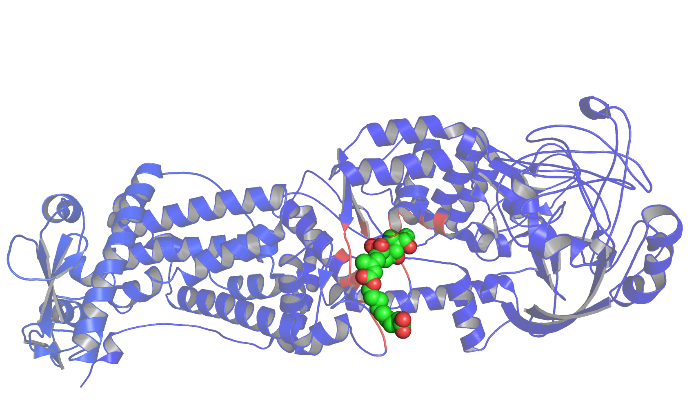

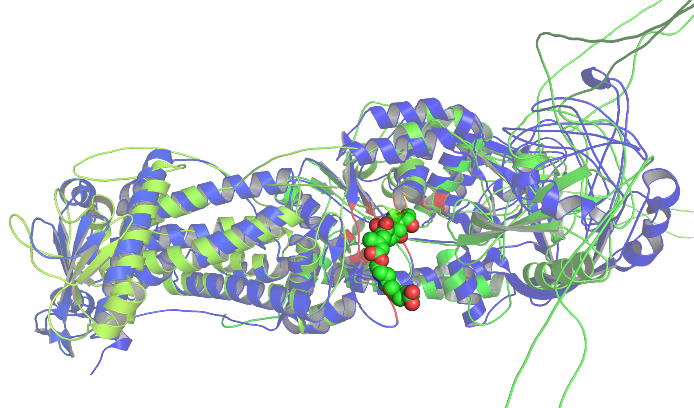

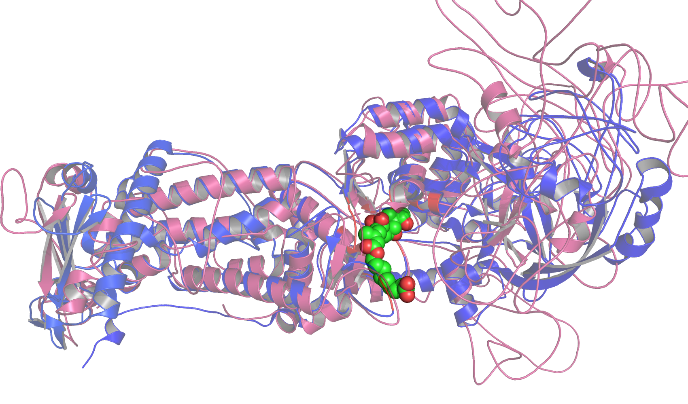


PF3D7_0622800

RMSD: 2.74

PF3D7_0311200

RMSD: 2.50

c)

b)

a)

1FFY


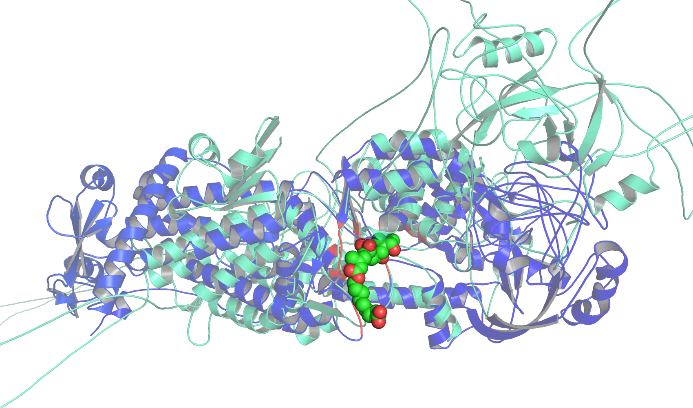

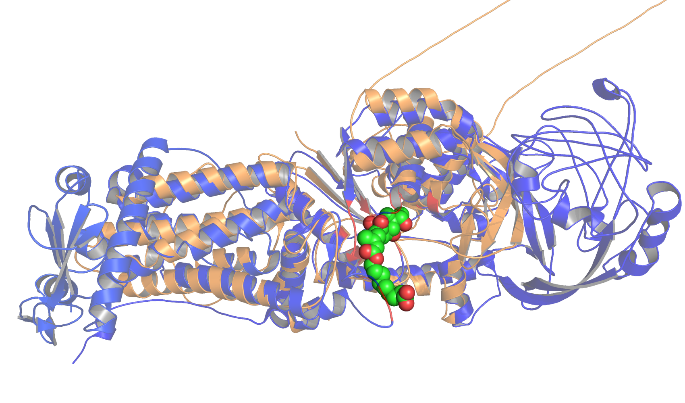

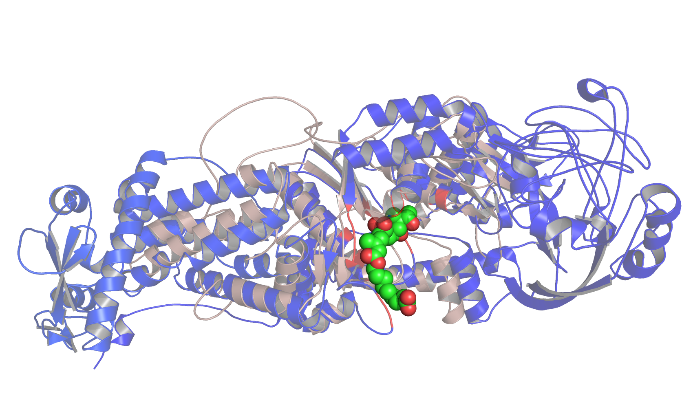


PF3D7_1015200

RMSD: 2.30

PF3D7_1005000

RMSD: 0.91

PF3D7_0828200

RMSD: 3.20

f)

e)

d)


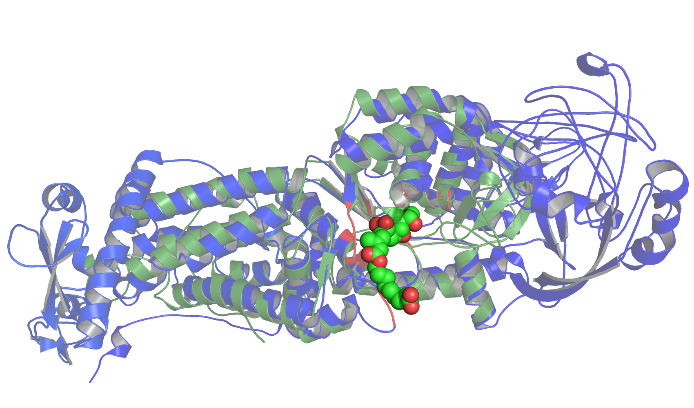

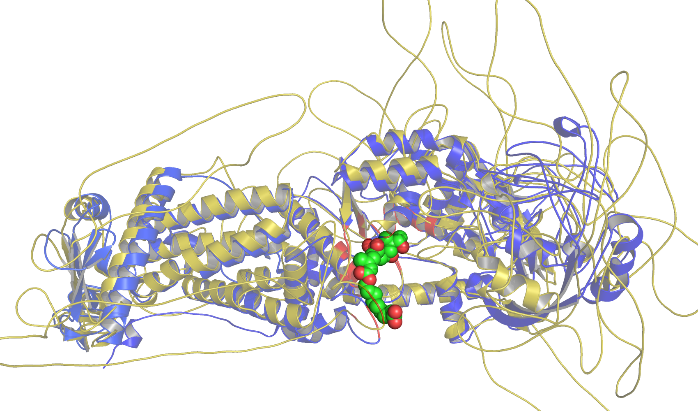

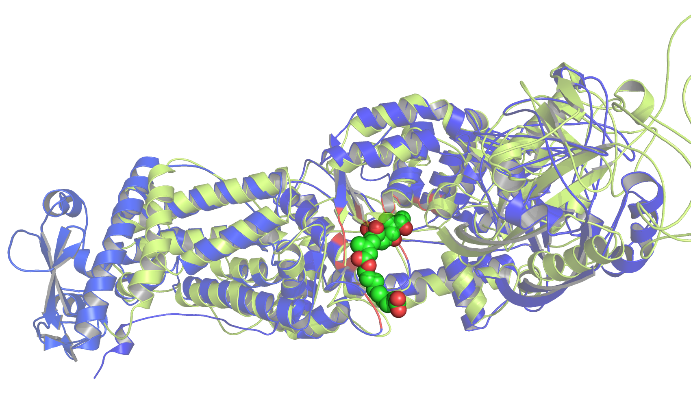


PF3D7_1332900

RMSD: 2.80

PF3D7_1225100

RMSD: 0.60

PF3D7_1034900

RMSD: 2.82

i)

h)

g)


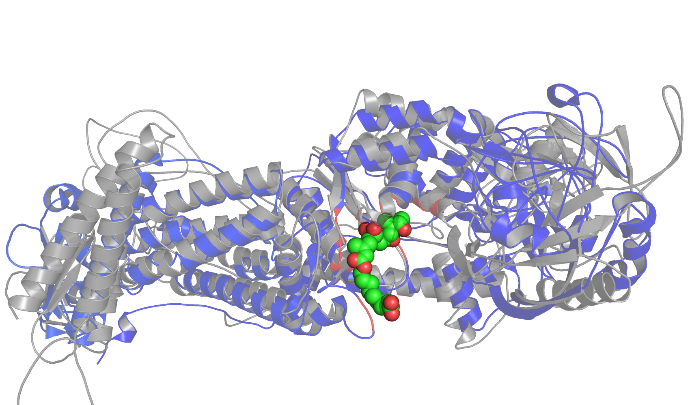


PF3D7_1461900

RMSD: 3.38

j)

Figure S1: Structural similarity between mupirocin-bound isoleucyl-tRNA synthetase and *P. falciparum* proteins. Modelled structures of nine *P. falciparum* proteins superposed on crystal structure of mupirocin-bound isoleucyl-tRNA synthetase [PDB ID: 1FFY] (deep blue) (a) are illustrated (b-j). The ligand mupirocin is represented as spheres and the residues around binding site in the target structure are coloured red in a). In the figures (b-j), except f) and g), the modelled structures of *P. falciparum* proteins are visibly ladened with long unstructured regions. These regions correspond to non-conserved inserts and/or low-complexity regions of the protein, which do not significantly influence the drug-binding site. The local RMSD achieved for each of the pairwise structural alignment is indicated.
